# Expressions of apoptotic protein and gene following sulfur mustard-induced acute pulmonary injuries in rats 

**DOI:** 10.22038/ijbms.2025.86449.18678

**Published:** 2025

**Authors:** Tao Liu, Jingtong Li, Xiaoxuan Hu, Jinyuan Tang, Yuxu Zhong, Xin Shu, Xiao-ji Zhu

**Affiliations:** 1 Department of Respiration, the 80th Group Army Hospital of People’s Liberation Army, Weifang, 261021, China; 2 Department of Pulmonary and Critical Care Medicine, Weifang No. 2 People’s Hospital, Weifang Respiratory Disease Hospital, Weifang, 261041, China; 3 State Key Laboratory of Antitoxic Drugs and Toxicology, Institute of Toxicology and Pharmacology, Academy of Military Medical Sciences, Beijing, 100850, China; 4 Department of Dermatology, The Third Medical Center of Chinese PLA General Hospital, Beijing, 100039, China

**Keywords:** Animals, Apoptosis, Immunohistochemistry, Lung injury, Mustard gas, Polymerase chain reaction

## Abstract

**Objective(s):**

Pathomechanisms of sulfur mustard (SM) are not fully understood, and no specific medical countermeasures exist to prevent SM-induced pulmonary injury. This study aimed to evaluate the apoptosis following SM-induced acute pulmonary injury.

**Materials and Methods:**

Acute pulmonary injury models were established using SM at an equivalent toxicity dose (1 LD50), administered via intraperitoneal injection or intratracheal instillation. Protein expression levels and mRNA expressions of apoptosis-related markers, including cellular inhibitor of apoptosis proteins-1 and -2 (cIAP-1, cIAP-2), Fas, Bcl-2-associated death promoter (Bad), second mitochondria-derived activator of caspases (Smac), and survivin (BIRC5), were analyzed using immunohistochemistry and polymerase chain reaction.

**Results:**

The intraperitoneal SM group exhibited significantly higher levels of apoptotic cells in the alveolar septa and increased protein and mRNA expression of cIAP-1, cIAP-2, Fas, Bad, Smac, and BIRC5 compared to the intratracheal SM group. These changes displayed a time-dependent increase in both protein and gene expression levels.

**Conclusion:**

SM-induced pulmonary injury involves both extrinsic (Fas, cIAP-1, cIAP-2) and intrinsic (Bad, Smac) pathways as well as caspase-dependent pathways (BIRC5). These findings provide valuable insights into the underlying mechanisms of SM toxicity and may facilitate the development of targeted therapeutic strategies.

## Introduction

Sulfur mustard (SM), a chemical warfare agent first introduced during World War I, has been deployed in various conflicts, including those in Syria and Iraq (1). In recent years, SM has raised significant public safety concerns due to its high toxicity, strong permeability, and ease of synthesis, making it a potential tool for terrorism (2). SM exists as both a liquid and vapor, primarily affecting the eyes, lungs, and skin. Notably, initial exposure often goes unnoticed, as symptoms typically appear only 10 minutes after ocular exposure or several hours following skin contact (3).

At the molecular level, SM acts as an electrophilic, bifunctional alkylating agent that readily reacts with cellular components, including lipids, proteins, and nucleic acids. DNA damage, particularly the alkylation of guanine nucleotides, leads to the formation of mono- and di-adducts and intra- and inter-strand crosslinks, contributing to its cytotoxicity (4). Other mechanisms, such as oxidative-nitrosative stress and elevated intracellular calcium levels ([Ca²⁺]i), exacerbate its toxic effects by triggering inflammatory responses and various forms of cell death (5, 6). For instance, the up-regulation of gelatinase MMP-9 in lung tissue following SM exposure promotes pro-inflammatory pathways and degrades extracellular matrix components, aggravating disease progression (7). Additionally, post-transcriptional epigenetic modifications, including increased levels of non-coding microRNAs, have been observed in both human and animal models following exposure to SM and nitrogen mustard (8).

Animal models have demonstrated that SM-induced pulmonary injuries can occur through diverse exposure routes, including percutaneous, subcutaneous, intraperitoneal, and oral administration. Remarkably, pulmonary toxicity has been observed even when the lungs were not directly exposed to SM, underscoring the systemic nature of its effects (9, 10). While inhalation appears to be the most relevant exposure route, nose-only inhalation in rats primarily causes upper airway injury (e.g., acute sclerosing nasopharyngitis), with minimal damage to the pulmonary airways or lung parenchyma due to the highly reactive nature of SM vapor and the rat’s extensive nasal passages. Significant tracheobronchial or distal airway damage occurs only when SM vapor is directly introduced into the trachea via a cannula (11). Consequently, intratracheal instillation is widely used for studying SM-induced pulmonary toxicities, offering a valid method for risk assessment (12). Similarly, intraperitoneal injection is another viable exposure route for investigating SM toxicity, though it does not perfectly replicate human exposure conditions (13).

Apoptosis, a regulated form of cell death, can occur through extrinsic (death receptor-dependent) or intrinsic (mitochondria-dependent) pathways. The extrinsic pathway involves the activation of death receptors on the cell surface by tumor necrosis factor (TNF) family ligands, while the intrinsic pathway is triggered by intracellular stress signals targeting mitochondria (14). Both pathways ultimately activate effector caspases, leading to morphological and biochemical changes characteristic of apoptosis. Apoptosis is intricately linked to other processes, such as oxidative stress, and plays a critical role in the pathogenesis of SM-induced pulmonary injuries. It also facilitates the clearance of neutrophils from sites of inflammation, with apoptosis predominating in regions of oxidative stress within the lungs (15). 

This study aimed to investigate whether SM induces apoptosis in acute pulmonary injuries and to evaluate differences in apoptotic cell levels in the alveolar septa of rats exposed to SM via intraperitoneal injection or intratracheal instillation. The results from both groups were then analyzed to identify any significant differences. The study findings can contribute to the designing of a new and effective therapeutic protocol.

## Materials and Methods

### Reagents and instruments

SM (CAS#: 505-60-2; purity 99%) was provided by the Institute of Toxicology and Pharmacology, Academy of Military Medical Sciences (Beijing, China); absolute ethanol solution was provided by Shanghai Aladdin Biochemical Technology Co., Ltd. (Cat # E130059; Shanghai, China); Goat anti-mouse/rabbit IgG polymer was provided by Zhongshan Jinqiao Biotech Co., Ltd. (Cat # ZF0314; Beijing, China; diluted in 1:100); Phosphate-buffered solution and the microwave buffer solution were provided by Chemical Plant, (Beijing, China); Cellular inhibitor of apoptosis proteins-1(cIAP-1) and second mitochondria-derived activator of caspases (Smac) immunohistochemical kits were provided by Abcam Co., Ltd. (Cat # ad2399; ad32023; Cambridge, MA, USA; diluted in 1:50); Factor-associated suicide (Fas) and Bcl-2 associated death promoter (Bad) immunohistochemical kits were provided by Solarbio Co., Ltd. (Cat # K000322P; K002861P; Beijing, China; diluted in 1:50); Cellular inhibitor of apoptosis proteins -2 (cIAP-2) and survivin (BIRC5) immunohistochemical kits were provided by Novus Biologicals Co., Ltd. (Cat # NBP1-90132, BN500-201; St. Louis, Missouri, USA; diluted in 1:50); SYBR Prime Script RT-PCR Kit II and the designed and synthesized primer sequences were provided by Solarbio Co., Ltd.; the quantitative fluorescence PCR instrument was purchased from Bio-Rad IQ5, Bio-Rad (Hercules, CA, USA), a cold light source (Gree Inc. Durham, North Carolina, USA), and the Pannoramic P250 was purchased from 3DHIESTECH Co., Ltd. (Budapest, Hungary).

### Ethical approval

The experiment was conducted under the guidance of Certifed Professional Institutional Animal Care and Use Committees (IACUC) Administrator (16). All carried out methods comply with Animals in Research: Reporting *In Vivo* Experiments (ARRIVE) guidelines (17). All experimental protocols were approved by the Animal Research Ethics Committee of the Institute of Toxicology and Pharmacology, Academy of Military Medical Sciences, Beijing, China (JSYXYJY-2023-1148, 06/18/2023).

### Animals approval 

A total of 236 male Sprague—Dawley rats (age: 15 weeks, weighing 280–300 g, specific pathogen-free) were purchased from the Laboratory Animal Center, Academy of Military Medical Sciences (Certification No. 0015902, Beijing, China). Of these 136 rats were randomly assigned to the following treatment groups of 32 rats each: intraperitoneal SM, intraperitoneal absolute ethanol control, intratracheal SM, and intratracheal absolute ethanol control; Untreated control group (8 rats). SM was diluted in absolute ethanol for subsequent use. The proportions of SM and absolute ethanol were SM (0.25 mg) in absolute ethanol (100 μl).

i. The establishment of intratracheal instillation animal model: The animals were housed in a separate room on a 12-hr light/12-hr dark cycle, with a temperature and humidity of 24 ± 2 degrees Celsius and 60–65%, respectively. The rats received a standard diet of food and water. Each rat was subcutaneously injected with atropine (0.05 mg/kg), followed by intraperitoneal administration of ketamine hydrochloride (100 mg/kg) 30 min later for anesthesia. The rats were then fixed in the supine position on the platforms so that the cold light source illuminated the necks of the rats. The epiglottises were elevated to expose the glottises using a self-made “L” hook. A 16# trocar (shaped to a blunt tip without a stylet) was placed into trachea, and then a fine plastic pipe was inserted into the trocar to complete the intubation. In the intratracheal SM group, each rat was instilled with 0.1 ml of the SM solution (0.98 LD_50_ = 2 mg/kg) into the trachea. The intratracheal absolute ethanol control group received 0.1 ml of the absolute ethanol solution via intratracheal instillation (12).

ii. The establishment of intraperitoneal injection animal model: The anesthetic method for the rats was the same as described earlier. In the intraperitoneal SM group, SM (0.1 ml/rat [0.96 LD_50_ = 8 mg/kg]) was injected intraperitoneally, while the intraperitoneal absolute ethanol control group received 0.1 ml of absolute ethanol intraperitoneally. Untreated control group was not treated with any substances (18). 

At the onset of the experiments, an acute pulmonary injury model was established via intraperitoneal injection and intratracheal instillation of SM in 100 rats, with the aim of calculating an equal toxicity dose (1LD_50_) using Horn’s method (19).

### Experimental methods

*Animal euthanasia and specimen collection*

At 6, 24, 48, and 72 hr after the last SM exposure, euthanasia of experimental rats was carried out by intraperitoneal injection of sodium pentobarbital (150 mg/kg). After administering the drug at 15–30 min, the breathing and heartbeat of experimental rats were stopped. The lung (right lower lobe) of rats designated for biochemical evaluations was removed. The lung tissue specimen was fixed in 10% formaldehyde, dehydrated in alcohol series, cleared in xylene, and embedded in paraffin, then serially sliced into 4-μm-thick sections. One section of each sample was used for hematoxylin and eosin staining. Each specimen was cut into 15 sections, with five sections per group. Immunohistochemistry of the sections were performed. In addition, fresh specimens were collected and immediately inserted into EP tubes, and then they were preserved in liquid nitrogen until used for PCR.

*Immunohistochemistry immunolabeling*

The streptavidin-biotin-peroxidase complex method (SP method) was used to detect the expressions of cIAP-1, cIAP-2, Bad, Fas, Smac, and BIRC5 proteins. The tissue samples were paraffin-embedded, sectioned, and deparaffinized with xylene, followed by antigen retrieval. Rabbit anti-rat monoclonal antibodies against cIAP-1, cIAP-2, Bad, Fas, Smac, and BIRC5 (20 μl/section) were added and incubated overnight at 4 °C. After incubation with the secondary antibody, the color was developed using diaminobenzidine (DAB), followed by hematoxylin counterstaining, routine dehydration, drying, and resin sealing. The primary monoclonal antibodies (cIAP-1, cIAP-2, Bad, Fas, Smac, and BIRC5) were diluted. The secondary antibody (goat anti-mouse/rabbit IgG polymer) was diluted with phosphate-buffered saline (PBS) containing 1% bovine serum albumin and 0.1% Triton solution. The concentration of DAB solution was used at 3.5%. PBS was used instead of the primary antibody for the negative control.

*Fluorescence quantitative PCR detection of cIAP-1, cIAP-2, Bad, Fas, Smac, and BIRC5 mRNA expression* Lung tissues from each group were used to extract the total RNA by using the Trizol reagent kit (Invitrogen, Carlsbad, CA, USA) in accordance with the manufacturer’s instructions. The total RNA was treated with DEPC ultrapure water (RNase-free) and stored at -80 °C. SYBR Green fluorescence quantitative PCR was used to detect the mRNA expression. The relevant primer sequences, the housekeeping gene β-actin (internal control), and the negative control prepared with ultrapure water (RNase-free) are listed in Table 1. The amplification efficiency results of each group were analyzed by using the 2^-^^ΔΔ^^Ct ^method.

*Microscopic image analysis*

Microscopic images of cIAP-1, cIAP-2, Bad, Fas, Smac, and BIRC5 proteins were analyzed by using Image Pro Plus 6.0 (Media Cybernetics, Rockville, MD, USA). For each group, specific measurement parameters were selected to evaluate the number of strongly positive and positive cells. Five high-power fields (×400) were observed in each section. The calculation of the proportion of positive cells in alveolar septal epithelial cells was as follows: positive cells/total cells in 5 high-power fields × 100%, and the average value of the positive cell ratio was obtained.

### Statistical analyses

Data were analyzed using SPSS 25.0 software (SPSS, Inc., IBM Corp., Armonk, NY, USA) and presented as the mean ±standard deviation. Two-way analysis of variance (ANOVA) was applied to assess the differences between the groups, wherever applicable. Dunnett’s post-test was used to evaluate the differences between the groups over time, with corrections for multiple comparisons. Apoptotic protein and gene analyses were used by two-way ANOVA, followed by the Student–Newman–Keuls multiple comparisons test. *P*<0.05 was considered to indicate statistical significance.

## Results

### Positive expression of cIAP-1, cIAP-2, Bad, Fas, Smac, and BIRC5 proteins 

**Figure 1:**
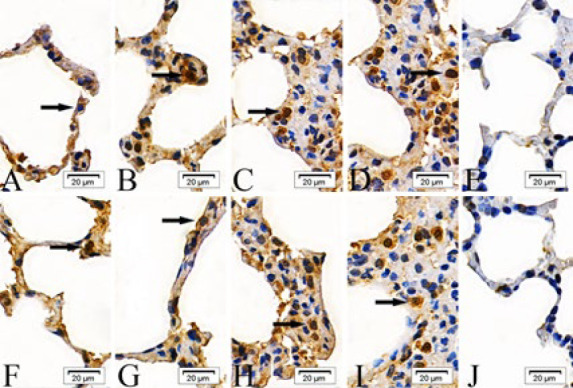
Expressions of cellular inhibitor of apoptosis proteins-1(cIAP-1) proteins in the epithelial cells of the alveolar septa in rats

**Figure 2:**
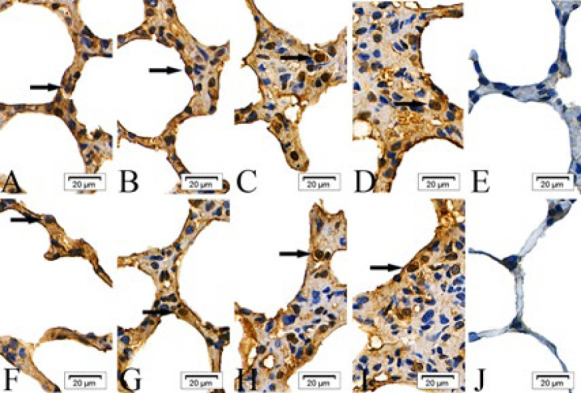
Expressions of cellular inhibitor of apoptosis proteins-2 (cIAP-2) proteins in the epithelial cells of the alveolar septa in rats

**Figure 3:**
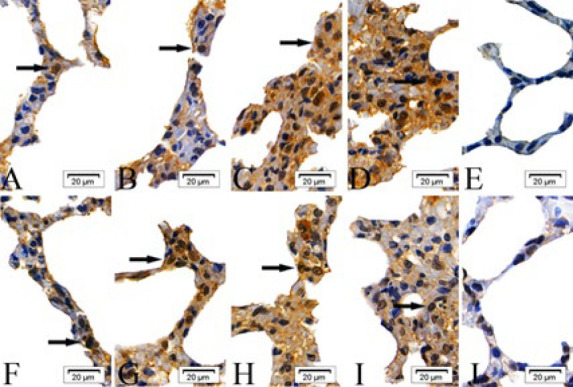
Expressions of Bcl-2 associtaed death promoter (Bad) proteins in the epithelial cells of the alveolar septa in rats

**Figure 4:**
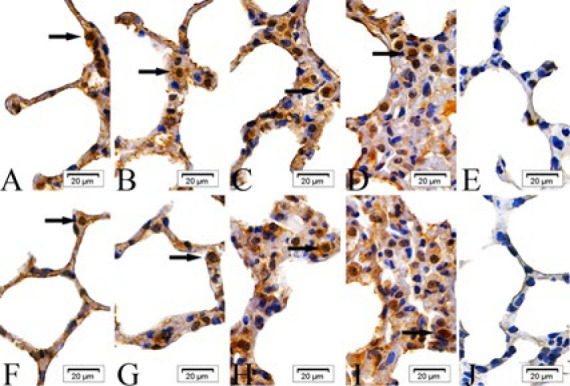
Expressions of factor associated suicide (Fas) proteins in the epithelial cells of the alveolar septa in rats

**Figure 5:**
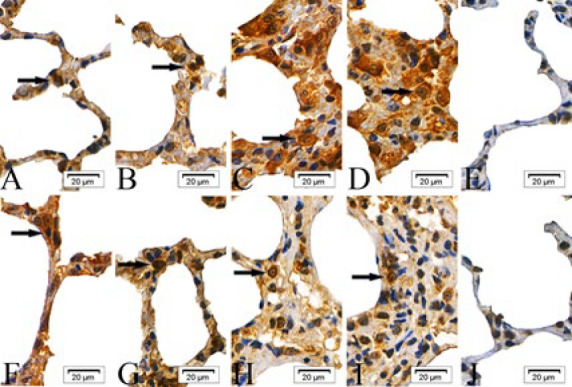
Expressions of second mitochondria-derived activator of caspases (Smac) proteins in the epithelial cells of the alveolar septa in rats

**Figure 6:**
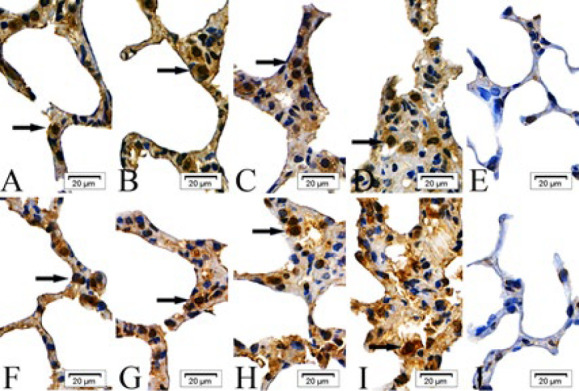
Expressions of survivin (BIRC5) proteins in the epithelial cells of the alveolar septa in rats

In the intraperitoneal and intratracheal SM groups, the expression of cIAP-1, cIAP-2, Bad, Fas, Smac, and BIRC5 proteins was scattered in the epithelial cells of the alveolar septa at 6 and 24 hr post-exposure. By 48 and 72 hr, these proteins were observed to form clusters. In contrast, the absolute ethanol control and control groups showed only scattered protein expression across all time points (Figures 1–6).

### Positive expression ratios of cIAP-1, cIAP-2, Bad, Fas, Smac, and BIRC5 proteins

**Figure 7:**
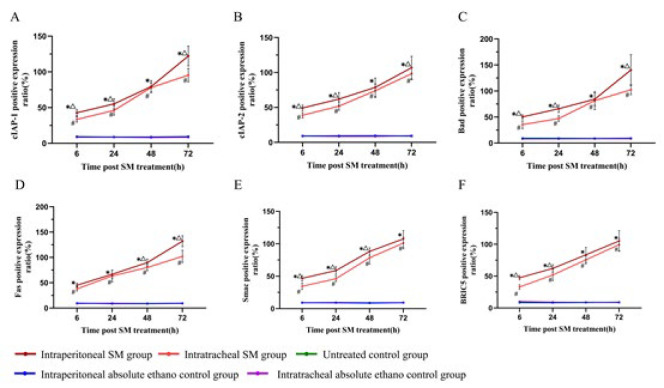
Positive expression ratios of cIAP-1(A), cIAP-2 (B), Bad (C), Fas (D), Smac (E), and BIRC5 (F) proteins in the epithelial cells of the alveolar septa in rats

The positive expression ratios of proteins in the epithelial cells of the alveolar septa were analyzed across five groups using repeated measures ANOVA:

i. At all-time points, both the intraperitoneal and intratracheal SM groups exhibited significantly increased positive expression ratios compared to controls.

ii. A statistically significant upward trend in protein expression ratios was observed over time in the intraperitoneal and intratracheal SM groups.

iii. The intraperitoneal SM group demonstrated a significantly higher positive expression ratio than the intraperitoneal and intratracheal absolute ethanol control groups and the control group.

iv. Similarly, the intratracheal SM group showed a significantly higher positive expression ratio than the control groups, with a progressive increase over time (Figures 7A-F).

### mRNA expression of cIAP-1, cIAP-2, Bad, Fas, Smac, and BIRC5

**Figure 8:**
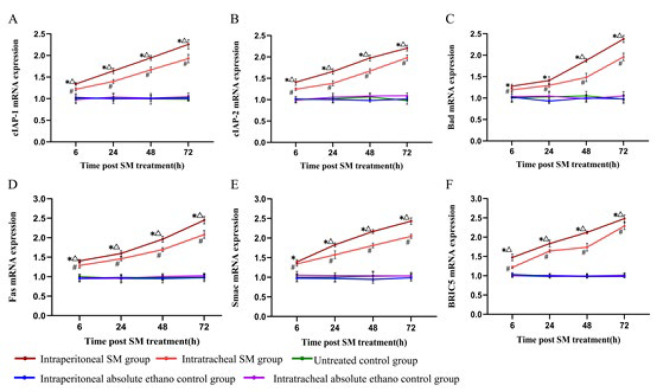
Messenger RNA (mRNA) expression of cIAP-1(A), cIAP-2 (B), Bad (C), Fas (D), Smac (E) , and BIRC5 (F) in the epithelial cells of the alveolar septa in rats

The mRNA expression levels in the epithelial cells of the alveolar septa were analyzed across five groups using repeated measures ANOVA at different time points, with the following observations:

i. At all-time points, the mRNA expression levels in the intraperitoneal SM and intratracheal SM groups were significantly elevated compared to the control groups.

ii. A statistically significant upward trend in mRNA expression was noted over time in both the intraperitoneal and intratracheal SM groups.

iii. The intraperitoneal SM group exhibited significantly higher mRNA expression levels than the intraperitoneal and intratracheal absolute ethanol control groups and the control group.

iv. Similarly, the intratracheal SM group showed markedly higher mRNA expression levels than the control groups, with a progressive increase observed over time (Figures 8A-F).

## Discussion

SM is a well-established inducer of apoptosis in the epithelial layers of the lung. Experimental evidence suggests that apoptosis is an early event following SM exposure, but a continuum from apoptosis to necrosis occurs depending on SM concentration and the time post-exposure (20). Apoptosis can occur via two primary pathways: the extrinsic (death receptor) and the intrinsic (mitochondrial) pathways, which are interconnected, allowing molecular cross-talk between them. Additionally, the perforin/granzyme pathway can induce apoptosis via granzyme B or granzyme A, initiating caspase-3 cleavage and resulting in DNA fragmentation, cytoskeletal and nuclear protein degradation, apoptotic body formation, and subsequent uptake by phagocytic cells. Granzyme A, in particular, activates a caspase-independent cell death pathway through single-stranded DNA damage (21). Moreover, endoplasmic reticulum (ER) stress plays a role in apoptosis via the unfolded protein response signaling pathway (22).

Apoptotic cell death and inflammation are closely linked to reactive oxygen species (ROS) generation. Preventing oxidative stress-induced tissue damage is a promising therapeutic approach for managing SM-induced injuries. Previous studies have highlighted the anti-inflammatory and anti-apoptotic effects of benzylisoquinoline alkaloid berberine (BER), which are attributed to its anti-oxidative properties (23). Oxidative stress links apoptosis and inflammation, suggesting synergistic therapeutic effects. BER’s anti-oxidative activity has been confirmed in several studies, which also demonstrated its modulation of oxidative markers and up-regulation of anti-oxidative protein expression through signal cascades like the Nrf2 pathway. Activation of the Nrf2 pathway may protect alveolar epithelial cells from SM-induced cytotoxicity (24).

Cellular inhibitors of apoptosis proteins, cIAP-1 and cIAP-2, play critical roles in suppressing apoptosis, and their up-regulation is associated with poor prognosis and disease progression. Inhibitors of apoptosis proteins (IAPs) exert anti-apoptotic effects primarily by inhibiting initiator and effector caspases directly or indirectly through ubiquitination, ultimately suppressing apoptosis (25). Additionally, cIAP-1 and cIAP-2 regulate anti-apoptotic activity via tumor necrosis factor-alpha (TNF-α)-mediated nuclear factor kappa B (NF-κB) signaling (26). The literatures also suggest that cIAP-1 can be cleaved by caspase-3 or other downstream caspases, such as caspase-8 via a feedback loop (27). 

Bad, a pro-apoptotic member of the Bcl-2 family, is inactivated by phosphorylation, which allows cells to evade apoptosis. In the absence of growth signals or glucose, Bad undergoes dephosphorylation, enabling it to interact with anti-apoptotic proteins such as Bcl-XL, Bcl-2, and Bcl-W. This interaction disrupts their inhibition of Bax and Bak, ultimately triggering apoptosis (28). Bad has also been shown to physically interact with p53, further promoting apoptosis (29). Activated Bad increases the permeability of the mitochondrial outer membrane, releasing pro-apoptotic proteins, including cytochrome c and Smac, into the cytosol. Cytochrome c then activates caspase-9, initiating the apoptotic cascade (30).

Fas is the most widely studied molecule among proteins of the death receptor family. The interaction between Fas and its ligand (FasL) is critical for inducing apoptosis. Cells expressing Fas undergo apoptosis upon interaction with FasL (31). Upon activation, Fas recruits the adapter molecule Fas-associated death domain (FADD) and caspase-8 to form the death-inducing signaling complex (DISC), which triggers the apoptotic cascade. Fas polarization has been associated with increased susceptibility to Fas-mediated apoptosis (32).

Smac is a novel pro-apoptotic protein released from mitochondria during apoptosis. Smac and cytochrome c are closely linked in mitochondrial apoptotic pathways (33). Smac promotes apoptosis through the mitochondria-mediated pathway by interacting with and inhibiting IAPs (34). Upon apoptotic signaling and the release of cytochrome c from mitochondria into the cytoplasm, Smac binds to the groove on XIAP-BIR3, displacing caspase-9 and relieving XIAP-mediated inhibition. 

BIRC5, a member of the apoptosis inhibitory protein family, plays a critical role in regulating apoptosis by influencing the expression of caspase proteins. It protects cells from apoptotic and autophagic death, with its cytoplasmic localization being essential for its anti-apoptotic activity, as nuclear translocation abrogates this function (35). The signaling mechanisms underlying BIRC5 biology are complex and context-dependent, with evidence suggesting that the activation of Akt/protein kinase B and phosphatidylinositol 3-kinase pathways occurs upstream of many BIRC5-mediated processes (36). Changes in BIRC5 expression are closely linked to neuronal apoptosis, with reduced expression leading to increased neuronal apoptosis, memory dysfunction, and the progression of neurodegenerative diseases (37).

Apoptotic cell death induced by SM or 2-chloroethyl ethyl sulfide (CEES) is associated with a series of complex biochemical and molecular events, along with specific structural changes in immunological cells. Sabnam *et al.* (38) demonstrated that CEES inhibited macrophage proliferation and induced apoptosis, as evidenced by DNA fragmentation, nuclear shrinkage, chromosomal shortening, and loss of chromosome number. Similarly, Steinritz *et al.* (39) reported that SM-induced apoptotic cell death is accompanied by a concentration-dependent increase in acetylcholinesterase activity, suggesting its involvement in regulating apoptosis. Furthermore, Mosayebzadeh *et al.* (40) utilized TUNEL assays and caspase-3 immunohistochemistry to detect apoptotic cells in SM-induced pulmonary injuries. Their results indicated significantly higher apoptosis in lung epithelial cells compared to controls, underscoring the role of apoptosis, inflammation, and oxidative stress as critical initiating factors, which may guide the search for anti-apoptotic targets.

*In vitro* and *in vivo* studies have shown that SM induces apoptosis (physiological cell death) and necrosis (pathological cell death) in a time- and dose-dependent manner (41). Both intrinsic and extrinsic pathways appear to contribute to SM-induced apoptosis (42). In our study, at equivalent toxicity doses, the positive expression ratios of apoptotic proteins and mRNA, including cIAP-1, cIAP-2, Bad, Fas, Smac, and BIRC5, were significantly higher in the epithelial cells of the alveolar septa in intraperitoneal SM groups compared to intratracheal SM groups at all time points. This difference may be attributed to the rapid absorption of SM through the peritoneal cavity. The mechanisms of apoptotic proteins and mRNA were as follows:

1. cIAP1 and cIAP2 inhibit caspase activity, particularly caspases-3, -7, and -9, and regulate apoptosis indirectly, especially at the level of death receptor signaling Instead of physically blocking caspase activity, they promote caspase degradation through ubiquitination and enhance cell survival by producing pro-inflammatory ubiquitin chains involved in inflammatory signaling pathways (43). Additionally, they influence ubiquitin-dependent pathways that activate NF-κB transcription factors and phosphatidylinositol-3 kinase signaling, which may concurrently mediate cIAP-2 up-regulation. In this study, we hypothesize that the increased protein and mRNA expression of cIAP-1 and cIAP-2 may represent a pathophysiological compensatory reaction aimed at maintaining the balance between pro-apoptotic and anti-apoptotic processes. 

2. The pro-apoptotic function of Bad is inactivated through phosphorylation. Dephosphorylated Bad dissociates from 14-3-3 proteins and translocates to the mitochondria, where it acts as an apoptotic inducer. This process leads to the release of cytochrome c, activation of caspase-9, and the initiation of apoptosis via the mitochondria-mediated pathway (44). Our findings align with this mechanism, as evidenced by the observed increase in Bad protein and mRNA expression.

3. Fas/FasL-mediated apoptosis is primarily regulated by the DISC, which includes FADD protein and aspartate-specific cysteine proteases such as caspase-8 and caspase-10. Fas and FasL, as pro-apoptotic proteins, mediate caspase-dependent cellular apoptosis (45). Our results confirm that SM-induced apoptosis in pulmonary injuries involves activation of the extrinsic pathway through ligand-activated death receptors like Fas/FasL. This finding is consistent with reports of a significant increase in soluble Fas (sFas) levels in the plasma of patients with severe chronic obstructive pulmonary disease. 

4. The translocation of mitochondrial pro-apoptotic proteins, such as Smac, is a critical regulatory mechanism for caspase activation. Smac may be released from mitochondria via the same or distinct pathways. Studies have shown that Smac and cytochrome c are co-released from mitochondria during ultraviolet light-induced apoptosis via a shared permeability transition mechanism, upstream of caspase-9 and caspase-3 activation. Overexpression of Smac enhances caspase activity and apoptosis, as seen in PC-3 cells treated with TRAIL (46). Our results corroborate these findings, demonstrating that Smac acts as a pro-apoptotic protein with a significant role in regulating cell apoptosis.

5. BIRC5 inhibits apoptosis via caspase-independent and caspase-dependent pathways. Survivin, the protein encoded by BIRC5, suppresses apoptosis by directly or indirectly inhibiting caspase activity, the key mediator of cell death. Overexpression of BIRC5 has been linked to the suppression of both intrinsic and extrinsic apoptotic pathways, with a stronger inhibitory effect on mitochondrial apoptosis compared to death receptor-mediated apoptosis (47). BIRC5 expression is transcriptionally up-regulated by NF-κB, which can be indirectly activated by growth factors via the phosphatidylinositol 3-kinase/Akt signaling pathway (48). In our study, the overexpression of BIRC5 protein and mRNA suggests its pivotal role in maintaining the dynamic balance between pro-apoptotic and anti-apoptotic factors.

On the whole, our study found that apoptosis-related proteins and apoptosis-related genes of the rats in the alveolar septa increases over time, and the number of apoptotic cells was positively correlated with the thickening of the alveolar septa, clinical ARDS, and mortality. In this study, the change trend of apoptosis are broadly consistent with the results of inflammatory response and oxidative stress in our previous study (49). Our findings also confirm that SM triggers apoptotic pathways (including intrinsic pathway, such as dephosphorylated Bad can lead to the release of cytochrome c, activation of caspase-9, and the initiation of apoptosis via the mitochondria-mediated pathway; Smac and cytochrome c are co-released from mitochondria via a shared permeability transition mechanism, upstream of caspase-9 and caspase-3 activation; extrinsic pathway, such as Fas-mediated apoptosis is regulated by the DISC, which includes FADD protein and aspartate-specific cysteine proteases, especially caspase-8 and -10, mediate caspase-dependent apoptosis: cIAP-1 and cIAP-2 modulate indirectly caspase activity and apoptosis at the level of death receptor signaling, and caspase-dependent pathways, such as BIRC5 can inhibit apoptosis via caspase-independent and caspase-dependent pathways) in the pathogenesis of pulmonary injury.

Our study also demonstrated a significant increase in the expression of apoptotic proteins and genes—including cIAP-1, cIAP-2, Bad, Fas, Samc, and BIRC5—in the alveolar septa of both intratracheal and intraperitoneal SM-exposed groups. This finding suggests that apoptosis plays a key role in the pathogenesis of SM-induced pulmonary injury. Our previous work has likewise confirmed the presence of apoptosis in this context (50). A study has reported that apoptosis contributes substantially to tissue damage during pulmonary injury (51). For instance, Kan *et al.* (52) highlighted apoptosis as one of the potential mechanisms underlying SM-induced lung damage. According to other reports, SM exposure can activate both mitochondrial and death receptor apoptotic pathways, as observed in keratinocytes (53,54). In pulmonary A549 cells exposed to SM, increased numbers of TUNEL-positive cells and cleavage of PARP were observed, indicating activation of effector caspases-3/7 (55). Ghazanfari *et al.* (56) also reported significantly elevated levels of serum soluble FasL in SM-exposed individuals with pulmonary complications compared to controls. Similarly, after SM exposure, Fas and FasL levels in bronchoalveolar lavage fluid increased markedly, although caspase-3 activity did not differ significantly between groups (57). Furthermore, Ray *et al.* (58) showed that SM induces apoptosis in cultured normal human bronchial/tracheal epithelial cells and small airway epithelial cells *in vitro*. Their findings support the involvement of a death receptor-mediated pathway, which may include a feedback amplification loop leading to caspase-9 activation via a caspase-3-dependent mechanism. It is also important to note that the detection of apoptotic features is time-sensitive, as apoptosis may proceed from initiation to cell death within a few hours (59). Taken together, these findings support the conclusion that apoptosis is a major form of cell death following SM-induced lung injury. As such, this process represents a promising target for evaluating apoptosis inhibitors or potential therapeutic candidates to mitigate SM-related pulmonary damage.

**Table 1:** Related primer sequences and amplified fragment length of cellular inhibitor of apoptosis proteins-1(cIAP-1), cellular inhibitor of apoptosis proteins-1(cIAP-2), Bcl-2 associtaed death promoter (bad), factor associated suicide (Fas), second mitochondria-derived activator of caspases (Smac) and survivin (BIRC5)

Gene	Genebank accession	Primer	Amplified fragment length
cIAP-1 cIAP-2 bad Fas Smac BIRC5 β-actin	NM_021752.2 NM_023987.3 NM_022698.1 NM_030989.3 NM_00100292.1 NM_022274.1 NM_031144.3	Upstream：5´- CGACTCTACGCTATATGAACAC -3´ Downstream：5´- ACTACCAGATGACCACAAGG -3´ Upstream：5´- TGTGCGACAGAACATTAGGAG -3´ Downstream：5´- AGGGAATGAAAACGAGGGAC -3´ Upstream：5´- CAACACAGATGCGACAAAGC -3´ Downstream：5´- GATGGGAGCGGGTAGAATTC -3´ Upstream：5´- TCCGACTATACCACTATCCACTAC-3´ Downstream：5´- GCACAAACACGAACCTCAAAG -3´ Upstream：5´- CAACACAGATGCGACAAAGC -3´ Downstream：5´- GATGGGAGCGGGTAGAATTC -3´ Upstream：5´- GCCGCCTTGGTGTCTTAC -3´ Downstream：5´- AAGTCCAGGTCACAATAGAGC -3´ Upstream：5´- TAAGGCCAACCGTGAAAAGAT -3´ Downstream：5´- GGTACGACCAGAGGCATACA -3´	180 bp 137 bp 132 bp 149 bp 132 bp 139bp 109bp

The rats of the intratracheal sulfur mustard (SM) group were instilled intratracheally with diluted SM (1LD50 = 2 mg/kg) and the rats of the intraperitoneal SM group were injected intraperitoneally with diluted SM (1LD50 = 8 mg/kg). The presence of immunolabelling (arrow) at 6, 24, 48, and 72 hr in the intraperitoneal SM group (A–D); the presence of immunolabelling (arrow) at 6, 24, 48, and 72 hr in the intratracheal SM group (F–I). The absence of immunolabelling in the control group (E and J); the absence of immunolabelling in the intraperitoneal and intratracheal absolute ethanol control groups were the same as that in the control group (cIAP-1 immunolabeling, ×400, magniﬁcation, Scale bar = 20 μm).

See Figure 1 for the rat treatment protocol. The presence of immunolabelling (arrow) at 6, 24, 48, and 72 hr in the intraperitoneal SM group (A–D); the presence of immunolabelling (arrow) at 6, 24, 48, and 72 hr in the intratracheal SM group (F–I). The absence of immunolabelling in the control group (E and J); the absence of immunolabelling in the intraperitoneal and intratracheal absolute ethanol control groups were the same as that in the control group (Fas immunolabeling, ×400, magniﬁcation, Scale bar = 20 μm).

See Figure 1 for the rat treatment protocol. The presence of immunolabelling (arrow) at 6, 24, 48, and 72 hr in the intraperitoneal SM group (A–D); the presence of immunolabelling (arrow) at 6, 24, 48, and 72 hr in the intratracheal SM group (F–I). The absence of immunolabelling in the control group (E and J); the absence of immunolabelling in the intraperitoneal and intratracheal absolute ethanol control groups were the same as that in the control group (Smac immunolabeling, ×400, magniﬁcation, Scale bar = 20 μm).

See Figure 1 for the rat treatment protocol. The presence of immunolabelling (arrow) at 6, 24, 48, and 72 hr in the intraperitoneal SM group (A–D); The presence of immunolabelling (arrow) at 6, 24, 48, and 72 hr in the intratracheal SM group (F–I). The absence of immunolabelling in the control group (E and J); the absence of immunolabelling in the intraperitoneal and intratracheal absolute ethanol control groups were the same as that in the control group (cIAP-2 immunolabeling, ×400, magniﬁcation, Scale bar = 20 μm).

See Figure 1 for the rat treatment protocol. The presence of immunolabelling (arrow) at 6, 24, 48, and 72 hr in the intraperitoneal SM group (A–D); the presence of immunolabelling (arrow) at 6, 24, 48, and 72 hr in the intratracheal SM group (F–I). The absence of immunolabelling in the control group (E and J); the absence of immunolabelling in the intraperitoneal and intratracheal absolute ethanol control groups were the same as that in the control group (Bad immunolabeling, ×400, magniﬁcation, Scale bar = 20 μm).

See Figure 1 for the rat treatment protocol. The presence of immunolabelling (arrow) at 6, 24, 48, and 72 hr in the intraperitoneal SM group (A–D); the presence of immunolabelling (arrow) at 6, 24, 48, and 72 hr in the intratracheal SM group (F–I). The absence of immunolabelling in the control group (E and J); the absence of immunolabelling in the intraperitoneal and intratracheal absolute ethanol control groups were the same as that in the control group (BIRC5 immunolabeling, ×400, magniﬁcation, Scale bar = 20 μm).

See Figure 1 for the rat treatment protocol. x±s, n = 8. *P<0.05, when compared with the intraperitoneal absolute ethanol control group. #*P*<0.05, when compared with the intratracheal absolute ethanol control group. □ *P*<0.05, when compared with the tracheal SM group. Intraperitoneal and intratracheal absolute ethanol control groups when compared with the corresponding control group.

See Figure 1 for the rat treatment protocol. x±s, n = 8. *P<0.05, when compared with the intraperitoneal absolute ethanol control group. #*P*<0.05, when compared with the intratracheal absolute ethanol control group. *P*<0.05, when compared with the tracheal SM group. Intraperitoneal and intratracheal absolute ethanol control groups were compared with the corresponding control group.

## Conclusion

This study confirms our hypothesis that the apoptotic mechanisms underlying SM (1LD50)-induced acute pulmonary injuries differ in rats exposed via intraperitoneal and intratracheal routes. Our findings demonstrate that the expression levels of proteins and mRNA, including cIAP-1, cIAP-2, Bad, Fas, Smac, and BIRC5, were significantly elevated in the epithelial cells of the alveolar septa in the intraperitoneal SM group compared to the intratracheal SM group at all examined time points. These results suggest that SM-induced apoptosis involves both the extrinsic and intrinsic pathways. This evidence provides valuable insights that could inform the development of novel and effective therapeutic strategies.

## Data Availability

All data generated or analyzed during this study are included in this article.

## Declaration

During the preparation of this work, the author(s) used ChatGPT, an AI language model developed by OpenAI, to assist with language editing, and improving the readability of the manuscript. After using this tool, the author(s) reviewed and edited the content as needed and take full responsibility for the content of the publication.
